# Dietary Fiber, Gut Microbiota, and Metabolic Regulation—Current Status in Human Randomized Trials

**DOI:** 10.3390/nu12030859

**Published:** 2020-03-23

**Authors:** Mari C. W. Myhrstad, Hege Tunsjø, Colin Charnock, Vibeke H. Telle-Hansen

**Affiliations:** 1 Department of Nursing and Health Promotion, Faculty of Health Sciences, Oslo Metropolitan University, Postbox 4, St. Olavs plass, 0130 Oslo, Norway; mmyhrsta@oslomet.no; 2 Department of Life Sciences and Health, Faculty of Health Sciences, Oslo Metropolitan University, Postbox 4, St. Olavs plass, 0130 Oslo, Norway; hetu@oslomet.no (H.T.); colin@oslomet.no (C.C.)

**Keywords:** gut microbiota, fiber, metabolic regulation, glycemic regulation, lipid metabolism, dietary intervention

## Abstract

New knowledge about the gut microbiota and its interaction with the host’s metabolic regulation has emerged during the last few decades. Several factors may affect the composition of the gut microbiota, including dietary fiber. Dietary fiber is not hydrolyzed by human digestive enzymes, but it is acted upon by gut microbes, and metabolites like short-chain fatty acids are produced. The short-chain fatty acids may be absorbed into the circulation and affect metabolic regulation in the host or be a substrate for other microbes. Some studies have shown improved insulin sensitivity, weight regulation, and reduced inflammation with increases in gut-derived short-chain fatty acids, all of which may reduce the risk of developing metabolic diseases. To what extent a dietary intervention with fiber may affect the human gut microbiota and hence metabolic regulation, is however, currently not well described. The aim of the present review is to summarize recent research on human randomized, controlled intervention studies investigating the effect of dietary fiber on gut microbiota and metabolic regulation. Metabolic regulation is discussed with respect to markers relating to glycemic regulation and lipid metabolism. Taken together, the papers on which the current review is based, suggest that dietary fiber has the potential to change the gut microbiota and alter metabolic regulation. However, due to the heterogeneity of the studies, a firm conclusion describing the causal relationship between gut microbiota and metabolic regulation remains elusive.

## 1. Introduction

Metabolic diseases, like type 2 diabetes (T2D) and cardiovascular diseases (CVD) are among the most important public health challenges in the world today [1]. Major risk factors contributing to the development of these diseases are linked to overweight and obesity and an unhealthy diet [2,3]. Epidemiological studies have linked a high intake of fiber to a reduced risk of T2D and CVD [4,5]. Furthermore, dietary fiber is well known for beneficial metabolic effects through its contribution to the reduction of cholesterol levels, improved control of blood glucose levels, and better regulation of body weight [6]. Our knowledge of the gut microbiota and its interaction with the host’s metabolic regulation has increased greatly during the last few decades [7]. Inflammatory and metabolic changes induced by the gut microbiome are hypothesized to play a role in the development of metabolic diseases such as T2D, CVD, and obesity [8,9,10,11].

In humans, the gut microbiota has evolved through a symbiotic relationship with the host. It offers the host benefits through the protection it provides against pathogens. It also contributes by maintaining intestinal barrier integrity, contributing to nutrient production and by producing metabolites such as short-chain fatty acids (SCFA) [12,13]. The gut microbiota consists of Bacteria, Archaea, and Eukarya. The Bacteria are the most abundant gut microorganisms of which the phyla Bacteroidetes, Firmicutes and Actinobacteria dominate numerically [14,15]. Several members of these phyla have been linked to the development of metabolic diseases [7]. A balanced bacterial composition is important for maintaining intestinal immunity and homeostasis. An imbalance of the gut microbiota is referred to as dysbiosis and has metabolic consequences [16]. Interestingly, T2D, CVD, and obesity have been associated with gut dysbiosis [17,18]. Furthermore, dietary components, including fiber, may influence the bacterial composition and microbial-derived metabolites and thereby host metabolism [7,19].

**Figure 1:**
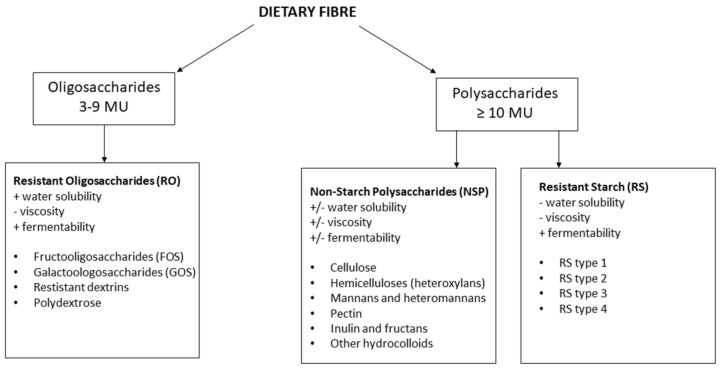
Classification of dietary fiber. NSP: Non-starch polysaccharides, MU: Monomeric units, RO: Resistant oligosaccharides, RS: Resistant starch.

Dietary fibers are either polysaccharides with a minimum of 10 monomeric units (MU)) or oligosaccharides containing between 3–9 MU. A further classification of dietary fiber is often based on their water solubility, viscosity, and fermentability [20,21,22]. Polysaccharides are further classified into non-starch polysaccharides (NSP) and resistant starch (RS), while oligosaccharides include resistant oligosaccharides (RO) as indicated in Figure 1. Furthermore, NSP, RS, and RO consist of several different fibers with different solubility, viscosity and fermentability characteristics. While RO in general are highly soluble and fermentable, but less viscous, RS are not soluble and viscous and only partly fermentable. NSP fibers vary to a large extent, with some being highly soluble, viscous, and fermentable while others are not [21]. Soluble fiber is typically fermented to SCFA, mainly acetate, propionate, and butyrate by the intestinal microbiota. Recent research has shown that SCFA have key roles in regulating host metabolism [19]. SCFA are transported into the systemic circulation and may directly affect host metabolism via binding to G-protein coupled receptors (GPR) [23,24]. These receptors are found to be expressed in several metabolically active tissues and are involved in responses to and regulation of many processes, including glucose homeostasis and lipid metabolism [25,26]. Evidence also suggests that SCFA may act as histone deacetylase inhibitors, thereby modulating gene expression [19]. Furthermore, SCFA are an important energy source for the intestinal epithelial cells and contribute to a strengthening of the gut barrier function [7,27]. Improved gut barrier function reduces the penetration of microbes and microbial molecules into the blood circulatory system, thereby reducing the immune responses that are associated with metabolic diseases [28]. There is also evidence that other microbial-produced metabolites may affect metabolic regulation. Indole and enterolactone are products of the microbial conversion of dietary tryptophan and lignan, respectively. Both are associated with increased fiber intake and a lower risk of T2D [29,30].

The evidence linking SCFA to metabolic regulation comes mainly from animal studies. Controlled human trials supporting the proposal of SCFA as key regulatory factors in human metabolism are largely lacking. Moreover, to what extent a dietary intervention with fiber may affect the human gut microbiota and production of SCFA, and hence metabolic regulation, is not well known. The aim of the present review is to summarize recent research on human randomized, controlled trials (RCT) investigating the effect of dietary fiber on gut microbiota and metabolic regulation.

## 2. Materials and Methods

**Figure 2:**
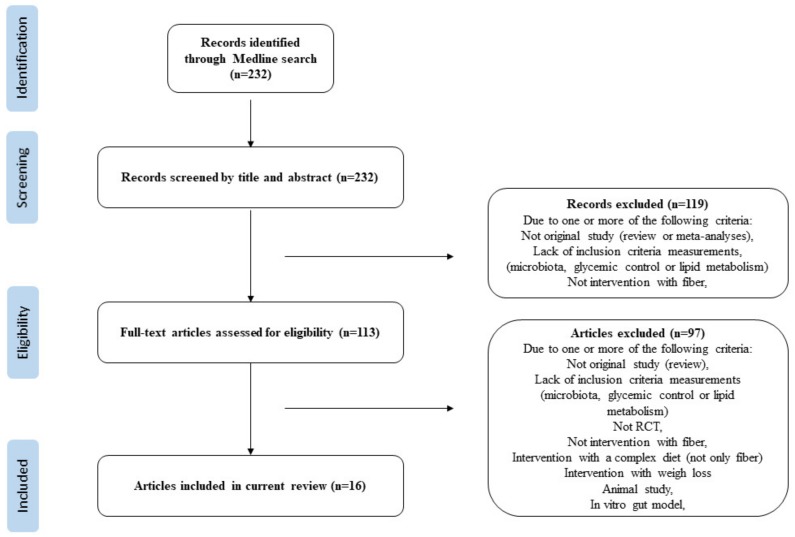
Flow chart showing the steps in the study selection.

To gain an overview of the effect of dietary fiber on gut microbiota and metabolic regulation, we performed a literature search in Ovid MEDLINE in September 2019. The following search terms were included: “gut microbiota or gastrointestinal microbiome” AND “fibre or fiber”. In addition, the search included “intervention or trials” performed in humans. The search resulted in 232 studies. Of these studies, only those, which clearly or possibly fulfilled the following criteria: metabolic regulation, gut microbiota, and RCT and intake of fiber were thereafter included. Metabolic regulation was defined as markers related to glycemic regulation such as glucose, insulin, glycolated hemoglobin A1c (HbA1c), homeostatic model assessment insulin resistance (HOMA-IR), Glucagon-like peptide-1 (GLP-1), C-peptide, and lipid metabolism such as total cholesterol (TC), low density lipoprotein-cholesterol (LDL-C), high density lipoprotein-cholesterol (HDL-C), triglycerides (TG), and non-esterified fatty acid (NEFA). We excluded studies that clearly fulfilled at least one of the following criteria: non-original studies (for example editorial, review or meta-analyses), studies that did not compare the criteria measurements to a control group, studies that did not report intake of fiber, studies that did not measure gut microbiota, animal studies, and studies that lacked inclusion criteria (as defined previously). In total, 16 articles were identified as eligible and these form the basis of the present review. Figure 2 summarizes in detail the study selection procedure in a PRISMA flow chart.

## 3. Results

Of 232 studies identified through the search strategy, only 16 studies had investigated the impact of dietary fiber on gut microbiota and host metabolic regulation with an RCT design. The studies were published between 2008 and 2019. The studies differed in the types of fiber given in the study design and in the duration of the intervention. In addition, the studies cover several different methodologies for the characterization of the microbiota. Both non-targeted and targeted DNA-based approaches were used, including metagenomics, 16S rRNA, quantitative polymerase chain reaction (qPCR), human intestinal tract chip (HITChip), and fluorescent in situ hybridization (FISH). Table 1 describes the different methods used for the microbiota analyses. Furthermore, the studies were conducted in healthy normal-weight individuals (Table 2, *n* = 5 studies), in overweight and obese (Table 3, *n* = 6 studies), and in people with metabolic diseases, such as T2D, Metabolic Syndrome (MetS), or Non-Alcoholic SteatoHepatitis (NASH) (Table 4, *n* = 5 studies). The studies are further described and presented below. The results of the microbiota and metabolic risk factors are reported as described by the authors in the original articles.

### 3.1. Healthy Normal-Weight Individuals

Five of the included studies have investigated the effect of fiber on gut microbiota and metabolic regulation in healthy, normal-weight individuals (Table 2). In a study by Sandberg et al., fecal samples from 99 individuals were analyzed, and the abundance of *Prevotella* and *Bacteroides* was quantified [31]. The analysis method for the microbiota was qPCR of 16S rRNA genes. Based on the baseline ratio of *Prevotella/Bacteroides,* a subset of the subjects was divided into three groups: high ratio group (*n* = 12), low ratio group (*n* = 13), and a group with a high abundance of both bacteria (*n* = 8). A short crossover intervention with barley kernel bread (total fiber intake 36.4 g/day) and white wheat bread (total fiber intake 10.7 g/day) was thereafter performed. Intake of barley lowered the blood glucose response in all three groups compared to white wheat bread, following a standard breakfast. The baseline levels of *Prevotella* and *Bacteroides* were not predictive of the metabolic response. Furthermore, the subjects with a high ratio of *Prevotella/Bacteroides* displayed a lower insulin response compared to the subjects with a low ratio, independent of the intervention [31]. A similar study was conducted by Kovatcheva-Datchary et al., where the intake of barley kernel bread was compared with white wheat bread in 39 healthy participants in a short-term crossover study [32]. The participants were classified as normal to slightly overweight based on body mass index (BMI). Postprandial blood glucose and serum insulin (after a standard breakfast) decreased after intervention with barley kernel bread compared to white wheat bread. Furthermore, from the total group, the authors selected for further study, ten subjects who showed little or no improvement in glucose and insulin responses (non-responders), and ten subjects who showed the most pronounced improvements in glucose and insulin responses. Analysis of the gut microbiota in these groups showed that responders had an elevated *Prevotella/Bacteroides* ratio and particularly an enrichment for the species *Prevotella copri*. The techniques used for the microbiota analyses were next-generation sequencing (NGS) of 16S rRNA genes and shotgun metagenomics. The metagenomics analysis revealed an increase in beta-glucan digesting enzymes after intervention in responders, which would be in line with increases in *Prevotella* species (spp). They also found decreased levels of postprandial blood glucose, the glucose incremental area under the curve (iAUC), and insulin in responders after intervention with barley kernel bread compared with white wheat bread [32]. The effect of whole-grain compared to refined grain was investigated by Vanegas et al. in a parallel design intervention study [33]. Eighty-one healthy men and women were given a diet containing either refined grain (reported daily intake of 21 g fiber) or whole-grain (reported daily intake of 40 g fiber). The authors describe changes in gut microbiota as revealed by NGS of 16S rRNA genes. Analyses were interpreted to the phylum and genus level (118 genera). Most noteworthy was a relative increase in the abundance of the Firmicutes genera *Lanchnospira* and *Roseburia* after intake of whole-grain compared to refined grain. Members of these genera are SCFA producers, and a positive correlation with stool acetate and butyrate levels was also found. In addition, the intake of whole-grain decreased the relative abundance of pro-inflammatory *Enterobacteriaceae*. Even though changes in the microbiota and stool SCFA were identified, no significant change in TG or cholesterol levels was found. A significant decrease in TC was, however, evident from baseline to post-intervention after intake of whole-grain [33]. Costabile et al., also investigated the effect of whole-grain wheat on gut microbiota compared to wheat bran [34]. Thirty-one healthy males and females were given breakfast cereals (48 g) with either whole-grain (11.8 g fiber/100 g) or wheat bran (27 g fiber/100 g) in a crossover study. After intervention with whole-grain cereals Bifidobacteria and Lactobacilli increased, whereas total bacteria, *Bacteroides* spp., Clostridia, *Atopobium* spp., *Bifidobacterium* spp., *Eubacterium rectale* group remained unchanged compared to the wheat bran intervention. Furthermore, intake of whole-grain increased the Lactobacilli/Enterococci ratio from baseline, and intake of wheat bran increased the abundance of *Bifidobacterium* spp. The chosen method of analysis was FISH, and probes targeting four major groups associated with intestinal health were used. No differences in glycemic regulation, markers of lipid metabolism, and fecal SCFA were observed. It is noteworthy to mention that the amount of fiber in the whole-grain cereal was lower than in wheat bran [34]. In another study by Costabile et al., the effects on gut microbiota and host metabolism were investigated after intake of probiotic strains combined with soluble corn fiber (SCF) [35]. The study was conducted in healthy, elderly persons who were given variously *Lactobacillus rhamnosus GG* + SCF; pilus-deficient *Lactobacillus rhamnosus GG-PB12* + SCF; SCF alone, or a control (maltodextrin) for 21 days. qPCR was used to quantify the *Lactobacillus rhamnosus* strains in fecal samples, and NGS of 16S rRNA genes was used for community analysis. In all intervention groups, the microbial composition changed slightly compared to the control diet, moving towards an increase in *Parabacteroides*. This increase was significant in the two symbiotic intervention groups. After intake of SCF alone, the concentrations of *Ruminococcaceae incertae sedis* was increased compared to the control diet. Compared to the control, there were no significant differences between treatments with respect to serum TC, HDL-C, LDL-C, TG, NEFA, or glucose. However, in participants with TC levels > 5 mmol/L, TC and LDL-C decreased from the baseline levels after intake of *Lactobacillus rhamnosus GG* + SCF [35].

In summary, in healthy, normal-weight people, the intake of fiber resulted in changes in gut microbiota in four of the five studies [32,33,34,35]. A concomitant beneficial change in the host metabolic factors was reported in three of these four studies [32,33,35]. A shift towards SCFA-producing strains and an increased ratio of *Prevotella/Bacteroides* after intake of whole-grain and barley may explain the observed effects on metabolic regulation.

### 3.2. Overweight and Obese Individuals

Six studies included in this review investigated the effect of fiber intake on gut microbiota and metabolic regulation in obese and overweight people (Table 3). In a crossover study by Kjølbæk et al., 27 overweight and obese participants were given 10.4 g/day arabinoxylan oligosaccharides (AXOS) or 3.6 g/day n-3 polyunsaturated fatty acids (PUFA) for twelve weeks [36]. After the AXOS intervention, the daily fiber intake was 31.2 g/day, and after the PUFA intervention, it was 20.9 g/day. The fiber was provided as wheat bran extracts enriched with AXOS. Analyses of gut microbiota were based on NGS of 16S rRNA genes and qPCR, which was used for absolute quantification of DNA molecules representing Bifidobacterium species. Intake of AXOS increased the abundance of several Bifidobacterium species compared to baseline. In addition, AXOS increased the relative abundance of butyrate-producing bacterial species. Beta-diversity analysis indicated that the structure of the gut microbiota only changed as a result of the AXOS intervention. There were no differences in gut microbiota after the PUFA intervention. Despite changes in the gut microbiota, glucose and lipid metabolic parameters did not change after any of the interventions [36]. A similar result was obtained after intake of galactooligosaccharides (GOS) in a study with a parallel design [37]. Canfora et al. performed a study with 44 overweight or obese prediabetic people. Participants were assigned to ingest 15 g/day GOS or placebo (matodextrin) with their regular meals for 12 weeks. Supplementation of the diet with GOS increased the abundance of Bifidobacterium species, whereas neither the microbial richness nor plasma SCFA were affected. Other taxa whose abundance was changed, although to a smaller degree, after intake of GOS compared to placebo were *Prevotella oralis, Prevotella melaninogenica, Bacteroides stercoris*, and *Stutterella wadsworthia*. Microbiota composition was analyzed with a phylogenetic microarray based on 16S rRNA gene sequences targeting more than 1000 intestinal bacteria. Even though a change in gut microbiota was observed, no significant alterations in fasting insulin, glucose, HOMA-IR, TG, or NEFA were observed after intake of GOS compared to placebo [37]. Chambers et al. investigated changes in glucose homeostasis, gut bacteria composition, plasma metabolome, and immune responses after intervention with the fructan inulin [38]. Twelve non-diabetic, overweight, and obese people participated in this crossover study, and these were given 20 g/day inulin or inulin-propionate ester. Inulin-propionate ester was given as a means to selectively deliver propionate to the colon. The study included two controls: one high in inulin (high fermentable fiber control) and one high in cellulose (low-fermentable fiber control). NGS of the 16S rRNA gene was the basis for microbiota analyses. Actinobacteria increased, and Clostridia and Clostridiales decreased in the group receiving inulin relative to the group receiving cellulose. At the species level, it was found that supplementation with both forms of inulin decreased the abundance of the selected species of Firmicutes and stimulated the growth of *Bacteroides* spp. It was also observed that inulin had a bifidogenic effect with an increased abundance of B. faecale compared with placebo. Furthermore, inulin-propionate ester compared to cellulose increased the level of propionate. Furthermore, both forms of inulin, compared with cellulose, reduced HOMA-IR, adipose tissue insulin resistance, and fasting insulin, and increased the Matsuda insulin sensitivity index [38]. In a study by Schuttle et al., the intake of whole-grain products (98 g/day) was compared to intake of refined wheat products (98 g/day) [39]. The study was conducted in 50 overweight and obese males and females and had a parallel study design. The α-diversity of the gut microbiota decreased, as revealed by NGS of 16S rRNA genes, in the refined wheat group compared to the whole-grain group. The authors focused on the effect of fiber on the Firmicutes families *Lachnospiraceae* and *Ruminovoccaceae*, and some commonly observed SCFA-producing genera belonging to these families. No significant differences between groups were found. Intake of whole-grain wheat compared to refined wheat did not affect fasting cholesterol, TG, NEFA, and insulin. Interestingly, the intrahepatic TG level was increased after the intake of refined wheat compared to whole-grain. However, the baseline microbiota composition could not predict the increase in intrahepatic TG after intake of refined wheat as assessed by machine learning [39]. Weickert et al. investigated whether diets rich in cereal-fiber improved insulin sensitivity via changes of gut microbiota in 69 overweight or obese people [40]. The study had a parallel design, and the participants received isoenergetic diets containing either high cereal-fiber (43 g/day), high-protein (28 E%/cereal-fiber 14 g/day), or moderately cereal-fiber and protein (23 E%/cereal-fiber 26 g/day) or control diet (cereal-fiber 14 g/day). None of the diets induced changes in gut microbiota and biomarkers of colonic fermentation, and fecal SCFA levels remained unchanged in all groups compared to the control. The gut microbiota analysis was performed with FISH coupled to the enumeration of taxa by flow cytometry, using both higher-order and species-specific probes. Even though no changes were observed in the gut microbiota, insulin-sensitivity increased within the high cereal-fiber group. The results indicate that the improvement of insulin sensitivity observed after intake of fiber was not related to changes in gut microbiota or markers of colonic fermentation [40]. Lambert et al. also conducted a parallel trial with 50 overweight and obese participants [41]. The participants were given isocaloric doses of pea fiber (15 g/day) or placebo wafers for 12 weeks. In line with the study by Weickert et al., no differences in gut microbiota between the groups were observed. The analysis method applied was qPCR with primers targeting genera (e.g., Bifidobacterium) and species (e.g., *Clostridium leptum*). They did not find any differences in fasting metabolic markers (HbA1c, TC, LDL-C, HDL-C, TG, TC:HDL) between the groups. However, during the OGTT, glucose AUC was lower in the pea fiber group at follow-up while insulin increased over time in both groups, but more so in the placebo group. It was concluded that the incorporation of 15 g yellow pea fiber per day might yield small but significant metabolic benefits without changing the gut microbiota [41].

In overweight and obese people, four out of six studies reported changes in gut microbiota after intake of fiber [36,37,38,39], and improved metabolic risk factors were found in half of these [38,39]. An increased bifidogenic effect and increased α-diversity of the gut microbiota were observed after intake of fiber. These changes may explain the described metabolic effects.

### 3.3. Individuals with Metabolic Related Disorders

Five of the studies covered in this review investigated the effect of fiber intake on gut microbiota and metabolic regulation in people with metabolic-related disorders (Table 4). In the study of De Faria Ghetti et al., 40 participants with NASH took part in a three months clinical trial [42]. The aim of the study was to investigate the effect of a fiber-rich diet on gut microbiota and metabolic regulation. The intervention group received a diet with 30 g/day of fiber (DIET group) in addition to nutrition orientation, whereas the control group received only nutritional orientation. The gut microbiota was analyzed with FISH, including phylum- and genus-level probes, and two additional probes targeting respectively *Escherichia coli* and *Clostridium histolyticum*. The study concluded that with regard to the fecal content of microbes, there was no significant difference between the groups at baseline or after three months of dietary intervention. However, the density of microorganisms increased within the DIET group. Within the control group, there was a reduction in Bacteroidetes and Verrucomicrobiales. Insulin, HOMA-IR, and TC decreased after intervention with fiber compared with the control group. In addition, glucose, insulin, HOMA-IR, TC and TG decreased significantly within the DIET group [42]. In people with T2D, Zhao et al. showed that a diet high in fiber altered gut microbiota and improved glucose homeostasis [44]. Twenty-seven participants received either usual dietary recommendations (Control; Chinese diabetes society guidelines) or a diet high in fiber in a parallel study design. The level of HbA1c, body weight, and blood lipid concentration decreased significantly after intake of the high-fiber diet. Selected strains of SCFA-producers were promoted by the intake of a high-fiber diet. These SCFA-producers harbored genes for acetate and butyrate production. In addition, when the fiber-promoted SCFA-producers were present in greater diversity and abundance, the effect on HbA1c was more prominent. After intake of dietary fiber enrichment of genes encoding Cohesin and Dockerin as part of a multi-enzyme complex for plant cell wall, degradation was also observed. The method used for the microbiota analysis was fecal shotgun metagenomics, providing both taxonomic and functional information on the complex microbial communities [44]. Velikonja et al. investigated the effect of the soluble fiber beta-glucan on alterations in the gut microbiota in a study with a parallel design [43]. Forty-three volunteers at risk or diagnosed with MetS consumed bread containing 6 g/day of barley beta-glucan (test group) or an equal amount of bread without beta-glucan (control group) for four weeks. The gut microbiota was analyzed with qPCR and NGS sequencing of 16S rRNA genes. Intake of beta-glucan reduced microbial diversity and richness compared to baseline. SCFA levels were altered with an increase in propionate after intake of beta-glucan and a decrease in acetate in the control group. After intervention, the plasma TC level decreased in the group receiving beta-glucan, but not in the control group. Interestingly, the cholesterol-lowering effect observed after intake of beta-glucan was associated with an increased abundance of *Bifidobacterium* spp. and *Akkermansia municiphilia* [43]. Connolly et al. also investigated the effect of beta-glucan on gut microbiota composition and metabolic regulation [45]. Thirty mildly hyper-cholesterolemic or glucose-intolerant males and females were given either 45 g/day non-whole-grain breakfast cereals with 3 g/day fiber and no detectable beta-glucan, or 45 g/day whole-grain oat granola with 6 g/day fiber and 2.9 g/day beta-glucan. The study had a crossover design. The total population of gut bacteria and the amount of bifidobacteria increased after the intervention period compared to the control period. In addition, during the intervention period, both the total bacteria population and the amount of bifidobacteria and lactobacilli increased. They measured gut microbiota using FISH probes, targeting a number of genera of importance for intestinal health. No differences in SCFA between the two periods were found. The study also reports a significant time by treatment interaction for TC and LDL-C after the intervention period compared to the control period. There were no differences in HDL-C, TG, and markers of glycemic regulation. The authors conclude that prebiotic modulation of the human gut microbiota may constitute a previously unrecognized mechanism contributing to the hyper-cholesterolemic effects of whole-grain oat granola rich in beta-glucan [45]. In a study by Pedersen et al., the effect of GOS intake on gut microbiota and glucose tolerance was investigated in 29 men with T2D [46]. The participants received either GOS (5.5 g/day) or placebo (maltodextrin) for 12 weeks. GOS supplementation had no significant effect on glucose tolerance outcomes or bacterial abundance. However, changes in the family *Veillonellaceae* correlated inversely with changes in glucose response following GOS intake. Gut microbiota was analyzed using high throughput NGS of 16S rRNA gene amplicons and by qPCR. The authors propose that the absence of significant changes to the microbial community under the study conditions is an important finding. However, the qPCR was restricted to a single species, *Clostridium leptum*, limiting the scope of the investigation [46].

In people with metabolic-related disorders, all of the presented studies reported a change in the gut microbiota after intervention with fiber [42,43,44,45,46]. A decrease in metabolic markers related to glycemic regulation or lipid metabolism was reported in four of the studies [42,43,44,45]. Both increased and decreased microbial diversity increases the SCFA-producers, and reduced abundance of Bacteroidetes was associated with a beneficial effect on metabolic regulation.

## 4. Discussion

The current review summarizes the impact of dietary fiber on gut microbiota and metabolic regulation in human RCT. Thirteen of the 16 studies included reported a change in gut microbiota, and a concomitant change in metabolic risk factors related to glycemic regulation and lipid metabolism was found in nine of these. Even though changes in both gut microbiota and metabolic biomarkers were found in several of the studies, the biological interpretation is complicated due to a myriad of methodological approaches employed.

The studies reviewed have mainly reported the effect of dietary fiber on gut microbiota by measuring the abundance of SCFA-producing bacteria, microbial diversity or richness, or the *Prevotella/Bacteroides* ratio. Several SCFA-producers were reported to be significantly increased after intake of dietary fiber, including *Lachnospira*, *Akkermannsia*, *Bifidobacterium*, *Lactobacillus*, *Ruminococcus*, *Roseburia*, *Clostridium*, *Faecalibacterium,* and *Dorea* [33,34,36,37,44,45]. This is in line with previous studies and indicates that certain types of soluble fibers, including inulin, beta-glucan, and GOS may increase the abundance of SCFA-producing strains [47,48,49,50]. Furthermore, increases in stool or plasma SCFA and a beneficial change in metabolic risk markers such as insulin sensitivity and cholesterol were evident after intake of refined grains, inulin, and beta-glucan [33,38,43]. SCFA derived from microbial fermentation of dietary fiber have recently been linked to beneficial effects on host metabolic regulation, via activation of G-coupled-receptors [19]. It is also interesting to note that in some studies, changes in microbial composition did not lead to changes in the SCFA level [34,37]. In line with this observation, not all fermentable fibers were able to increase butyrate production in a study performed in 174 healthy young adults. The authors discuss the importance of the individual microbiota composition in determining whether they will respond to a specific dietary supplement [51]. It is also relevant to consider the impact of functional redundancy in the gut; changes in the bacterial composition will not always translate into functional changes. Due to a limited number of studies reporting changes in SCFA and concomitant beneficial effects on metabolic regulation, the relation between dietary fiber, production of SCFA, and metabolic regulation requires further investigation.

In general, a shift towards higher diversity or richness in gut microbiota is considered healthy [52]. In the current review, increased diversity or richness and beneficial effects on metabolic risk factors were reported by Ghetti et al. and Shuttle et al. after intake of a diet rich in fiber or whole-grain [39,42]. Conversely, Zhao et al. and Velikonja et al. challenge the notion that greater overall diversity implies better health. Zhao et al. reported a reduction in gene richness and improved glycemic regulation after intake of a diet rich in fiber [44]. Furthermore, Velikonja et al. reported a reduction in overall diversity and cholesterol after dietary intervention with barley beta-glucan [43]. This may indicate that diet-induced metabolic responses are dependent on individual microbiota composition and the abundance of specific carbohydrate fermenting bacteria, rather than the overall microbial diversity. In line with this, Zhao et al. showed that the effect on HbA1c was more prominent where fiber-promoted SCFA-producers were present in greater diversity and abundance [44]. Furthermore, the cholesterol-lowering effect observed after intake of beta-glucan was associated with higher abundances of specific SCFA-producing species such as *Bifidobacterium* spp., and *Akkermansia municiphilia* [43].

A plant-based diet rich in fiber has recently been linked to a *Prevotella* enterotype, whereas the *Bacterioides* enterotype was associated with a high intake of protein and fat [53,54]. In the study by Sandberg et al. the *Prevotella/Bacteroides* ratio at baseline was not predictive of the metabolic response to grain intervention [31]. This was, with the exception of some participants, also the finding after intake of whole-grain in the study by Schuttle et al. [39]. Furthermore, Kovatcheva-Datchary reported improved glucose metabolism after intake of barley kernel bread in persons with an elevated *Prevotella/Bacteroides* ratio (responders) and particularly an enrichment for the species *Prevotella copri* [32]. The authors hypothesized that the increased ratio in the responders could be a consequence of a higher habitual fiber intake, which has been proposed to be associated with increased levels of *Prevotella* spp. [55]. Taken together, a diet high in fiber may be linked to a specific enterotype rich in *Prevotella*. However, whether this enterotype is associated with improved metabolic regulation needs to be investigated.

The methodological differences in microbiota studies are numerous and there are challenges to be addressed. Most of the studies reviewed in this paper have collected stool samples without additives and frozen the samples immediately or within three hours of collection, a procedure recommended to provide microbiome stability [56]. However, few studies have included information about homogenization prior to DNA extraction [33]. Sinha et al. have shown that variability in 16S rRNA targeted amplicon sequencing depends mainly on bio-specimen type and origin, followed by DNA extraction, sample handling environment, and bioinformatics [57]. Furthermore, previous studies have reported that both DNA extraction and PCR amplification contribute to the variability observed in studies using 16S rRNA amplicons [58]. The different regions of the 16S rRNA gene are variably informative and, perhaps more importantly, the different primers have different affinities to bacterial taxa. Few if any of the studies using 16S rRNA amplification have reported results from positive controls with known bacterial compositions. This would have allowed for better comparison between studies and would help to identify biases in different protocols. Discrimination at the species level is not always possible when only a small region of the 16S rRNA gene is sequenced. Therefore, the genus level is a widely used level for taxonomic comparison, limiting the scope of the information obtained. This is also generally the case for many of the studies on which this review is based. Other methods such as FISH, qPCR, and metagenomics have also been utilized in the studies included, and the use of different methods complicates the inter-study comparisons. Several of the studies utilizing methods such as FISH [42,45] or qPCR [41] have focused on predefined taxa and may, therefore, lack information on specific strains. Only two of the studies performed non-targeted shotgun metagenomics [32,44]. This method offers an advantage as it sequences all DNA in a sample and therefore defines taxonomic distribution to species and strain level. Furthermore, the characterization and quantification of the microbiota are not limited by primer bias, choice of variable regions, or PCR competition [59]. Non-targeted shotgun metagenomics will also identify functional bacterial genes. In the study by Kovatcheva-Datchary et al., the metagenomics analysis revealed an increase in beta-glucan digesting enzymes after intervention with beta-glucan in individuals responding to the fiber diet [32]. Zhao et al. showed an increase in SCFA related genes known to be involved in acetate and butyrate production after the intake of a diet rich in fiber [44]. A larger focus on standardization of protocols and quality controls needs to be addressed in order to adequately explore diet-related effects on gut microbiota. In addition, the use of non-targeted metagenomic approaches will achieve more information on microbiota functionality. Whereas most of the included papers documented changes in gut microbiota after intake of fiber, the effect on metabolic regulation was less prominent. Dietary fiber is well known for beneficial metabolic effects by reducing cholesterol levels and improving control of blood glucose levels [60,61]. In the current review, the effect on metabolic risk factors was more prominent in people with metabolic disorders than in the healthy study group. The effect of diet on circulating risk factors is, in general, small and difficult to measure in healthy people with risk factors within a normal range due to large inter-individually differences. The lack of effect on metabolic risk factors may also be related to study design and duration and the type of fiber given. Fiber is a large group of molecules with different health effects, and comparing different qualities, and quantities of fiber may explain the lack of clear results on both metabolic risk factors and gut microbiota. Furthermore, it has been suggested that the gut microbiota influences the host via a range of microbiota-derived metabolites [62,63]. A non-targeted metabolome approach may therefore offer a better and more precise mechanistic insight into the relationship between gut microbiota and host metabolic regulation.

## 5. Conclusions

The current review shows that dietary fiber has the potential to change the gut microbiota and alter metabolic regulation in humans. Although the current review indicates that the effects may be related to an increased abundance of SCFA-producers, alterations in microbiota diversity and the *Prevotella/Bacteroides* ratio, the interpretation is complicated due to differences in methodology. More studies providing both taxonomic and functional information on the microbial communities, in combination with untargeted metabolome analyses, would offer a broader understanding of gut microbiota and host metabolic regulation.

## Figures and Tables

**Table 1:** Methods used for microbiota analyses in the publications covered in the current review.

Methods		Principals	+/−
**Non-targeted DNA-based approach**	**Non-targeted shotgun metagenomics**	Sequences all DNA in a sample Defines taxonomic distribution to species and strain level Identifies functional genes and may assemble whole genomes	+ Not limited by primer bias, choice of variable regions or PCR competition − Less tolerant of low biomass − Less tolerant of contaminating DNA
**Targeted DNA-based approaches**	**16S rRNA/ITS amplicon massive parallel sequencing**	Amplifies and sequences regions that are present (but variable) in all bacteria Defines taxonomic distribution to genus level.	+ Can be performed on low biomass samples + Tolerates contaminating DNA − Limited by primer bias, choice of the variable region, PCR competition
	**Targeted quantitative PCR (qPCR)**	Quantifies selected genera/species using specific primers and fluorescently labeled probes	+ Not limited by PCR bias or competition + Can be performed on low biomass samples + Tolerates contaminating DNA − Only selected taxa are quantified
	**Microarray—HITChip** **(Human intestinal tract chip)**	Amplifies 16S rRNA regions that are present (but variable) in all bacteria, then hybridizes amplicons to microarray with probes	+ Can be performed on low biomass samples + Tolerates contaminating DNA − Limited by primer bias, choice of variable region, PCR competition − Only pre-defined taxa are identified
	**Fluorescent in situ hybridization (FISH)**	Quantifies selected taxa using specific fluorescently labelled DNA probes that hybridize to a fixed intact sample Sample is imaged, or flow cytometry is applied for cell counting	+ Can be performed on low biomass samples + Tolerates contaminating DNA − Limited by subjective measures, labor-intensive − Only selected taxa are quantified

+/−: advantages/disadvantages.

**Table 2:** Fiber, gut microbiota, and metabolic regulation in healthy individuals. Significant results are indicated by an up/down arrow.

Study	Subject Characteristics	Study Design	Intervention	Changes Related to Gut Microbiota	Changes Related to Metabolic Regulation
Sandberg et al., Eur J Nutr, 2019 [31]	*n* = 99, BMI 24, 64 year, M/F, stratified in 3 groups based on *Prevotella/Bacteroides* ratio + total group	2 × 3 days Crossover	(1) White wheat bread (fiber 10.7 g/day) (2) Barley kernel bread (fiber 36.4 g/day)	Stratified on *Prevotella/Bacteroides* ratio *Prevotella/Bacteroides* ratio was not predictive of the metabolic response.	↓ iAUC Glu (after barley kernel bread all groups) ↓ iAUC Ins (after barley kernel bread all groups) ↔ NEFA
Costabile et al., Front Immunol, 2017 [35]	*n* = 36, BMI 26–28, 60–80 year, M/F	4 × 21 days Crossover	(1) Maltodextrin (2) Soluble corn fiber (SCF) (8 g/day) (3) *Lactobacillus rhamnosus* GG + SCF (8 g/day) (4) Pilus-deficient *L. rhamnosus* GG-PB12 + SCF (8 g/day)	(3), (4) ↑ *Parabacteroides* (2), (3) ↑ *Ruminococcaceae incertae sedis* (3) ↓ *Oscillospira* (3), (4) ↓ *Desulfovibrio*	↔ Glu ↔ TC, LDL-C, HDL-C, TG, NEFA Within group: (3) ↓ TC, LDL-C (participants with TC > 5 mmol/L))
Vanegas et al., Am J Clin Nutr, 2017 [33]	*n* = 81, BMI 26, 55 year, M/F	6 weeks Parallel	(1) Refined-grain diet (fiber 8 g/1000 kcal) (2) Whole-grain diet (fiber 16 g/1000 kcal)	↔ Phylum level ↓ *Enterobacteriaceae* *↑ Lachnospira*, *Roseburia* Correlations: ↑ *Lachnospira* and *Roseburia* and acetate and butyrate ↑ SCFA (stool), acetate (stool)	↔ LDL-C, HDL-C, VLDL-C, TG Within group: (2) ↓ TC
Kovatcheva-Datchary et al., Cell Metab, 2015 [32]	*n* = 39, BMI 18–28, 50–70 year, M/F Responders, *n* = 10 Non-responders, *n* = 10	2 × 3 days Crossover	(1) White wheat bread (fiber 9.1 g/day) (2) Barley kernel bread (fiber 37.6 g/day)	Responders vs. non-responders: ↑ Bacteroidetes ↑ *Prevotella/Bacteroides* ratio	↓ Glu, Ins (postprandial) Responders vs. non-responders: ↓ iAUC Glu ↓ iAUC Ins
Costabile et al., Br J Nutr, 2008 [34]	*n* = 31, BMI 20–30, 25 year, M/F	2 × 3 weeks Crossover	(1) Wheat bran cereal, 48 g, breakfast (fiber 27 g/100 g) (2) 100% whole-grain cereal, 48 g, breakfast (fiber 11.8 g/100 g)	↑ Bifidobacteria, Lactobacilli, ↔ Total bacteria, *Bacteroides* spp., Clostridia, *Atopobium* spp., *Bifidobacterium* spp., Eubacterium rectale group ↔ Acetate, Butyrate, Caprionate, Propionate Within groups: (1), (2) ↑ Lactobacilli/Enterococci ratio (2) ↑ *Bifidobacterium* spp.	↔ Glu, Ins ↔ TC, TG, HDL-C

Significant differences (*p* ≤ 0.05) between the intervention group(s) and control group are shown with ↑ or ↓ while ↔ indicates no significant difference. When several intervention groups are present, the results for each group are indicated with the number. Within-group changes are indicated with numbers. Fasting values are shown, if not otherwise stated. The control group is referred to as (1). BMI: body mass index, F: Female, Glu: Glucose, g: gram, HbA1c: Glycated hemoglobin A1c, HOMA-IR: Homeostasis assessment model-insulin resistance, HDL-C: HDL-Cholesterol, iAUC: Incremental Area Under the Curve, Ins: Insulin, LDL-C: LDL-Cholesterol, M: Male, *n*: numbers, NEFA: Non-esterified fatty acids, TG: Triglycerides, TC: Total cholesterol.

**Table 3:** Fiber, gut microbiota, and metabolic regulation in overweight and obese individuals. Significant results are indicated by an up/down arrow.

Study	Subject Characteristics	Study Design	Intervention	Changes Related to Gut Microbiota	Changes Related to Metabolic Regulation
Chambers et al., Gut, 2019 [38]	*n* = 12, BMI 30, 60 yaer, M/F	3 × 42 days crossover	(1) High cellulose (20 g/day) (2) High inulin (20 g/day) (3) Inulin-propionate ester (IPE) (20 g/day)	IPE and inulin compared to cellulose: ↓ Diversity of bacterial species Inulin compared to cellulose: ↓ Enrichment (changes in evenness), ↔ Phyla level, ↑ Actinobacteria, *Anaerostipes hadrus*, *Bifidobacterium faecale*, *Bacteroides caccae ↓* Clostridia, Clostridiales, *Blautia obeum, Blautia luti, Oscillibacter* spp., *Blautia faecis*, *Ruminococcus faecis* IPE compared to cellulose: ↔ Phyla level,↑ *Bacteroides uniformis*, *Bacteroides xylanisolvens*, ↓ *Blautia obeum, Eubacterium ruminantium* ↑ Propionate (% in serum), ↔ propionate (uM in feces and serum, % in feces), acetate (% and uM in feces and serum), butyrate (% and uM in feces and serum) IPE compared to inulin: ↔ Phyla level, ↑ *Fusicatenibacter saccharivorans*, ↓ *Anaerostipes hadrus*, *Blautia faecale, Prevotelle copri*	IPE and Inulin compared with cellulose: ↓ Ins, HOMA-IR, AT-IR, ↑ Matsuda ISI
Kjølbæk et al., Clin Nutr, 2019 [36]	*n* = 27, BMI 25–40, 18–60 yaer, M/F	2 × 4 weeks Crossover	(1) n3 PUFA (3.6 g/day) (2) Arabinoxylan oligosaccharides (10.4 g/day)	Within groups: (2) in responders: ↑ Actinobacteria, *Eubacterium rectale*, *Faecalibacterium prusnitzii*, *Bifidobacterium faecale*, *Bifidobacterium stercoris*, *Bifidobacterium dolescentis*, *Blautia wexlerae*, *Bifidobacterium angulatum*, *Bifidobacterium merycicum*, *Bifidobacterium pseudocatenulatum*, *Bifidobacterium catenulatum*, *Fusicatenibacter saccharivorans*, *Bifidobacterium longum*, *Ruminococcus obeum*, *Dorea longgicaterna*, *Eubacterium hallii*, *Blautia luti* *↓ Clostridium methylpentosum*, *Anaerotruncus colihominis*, *Erysipelothrix rhusiopathiae*	↔ Glu, Ins, HOMA-IR, HOMA-β ↔ TC, HDL-C, LDL-C, VLDL-C, ApoB
Schutte et al., Am J Clin Nutr, 2018 [39]	*n* = 50, BMI 25–35, 61 year, M/F	12 weeks Parallel	(1) Refined wheat (98 g/day) (2) Whole-grain wheat (98 g/day)	↑ α-diversity, ↔ *Lachnospiraceae* and *Ruminovoccaceae* (and genera within these families)	↔ Glu, Ins, HOMA-IR ↔ TC, HDL-C, TG, NEFA ↓ IHTG
Canfora et al., Gastroenterology, 2017 [37]	*n* = 44, BMI 28–40, pre-diabetic, 45–70 year, M/F	12 weeks Parallel	(1) Maltodextrin (15 g/day) (2) Galacto oligosaccharides (15 g/day)	↑ *Bifidobacterium* spp., *Prevotella oralis*, *Prevotella melaninogenica* *↓ Bacteroides stercoris*, *Sutterella wadsworthia* ↔ Fecal microbial richness or diversity ↔ SCFA (fecal and plasma)	↔ Glu, Ins, HOMA-IR, GLP-1 ↔ TG, NEFA
Lambert et al., Clin Nutr, 2017 [41]	*n* = 50, BMI 33, 44 year, M/F	12 weeks Parallel	(1) Wafers without pea fiber (2) Wafers with pea fiber (15 g/day)	↔ Total bacteria, *Bacteroides/Prevotella* spp., *Bifidobacterium* spp., *Enterobacteriaceae*, *Methanobrevibacter* spp., Firmicutes, *Lactobacillus* spp., *Clostridium leptum* (C-IV), *Clostridium coccoides* (C-XIVa), *Clostridium* cluster I, *Clostridium* cluster XI, *Roseburia* spp.	↔ Glu, Ins, HbA1c ↔ TC, LDL-C, HDL-C, TG, TC/HDL-C ratio
Weickert et al., Nutr Metab, 2011 [40]	*n* = 69, BMI >30, 55.3 year, M/F	18 weeks, Parallel	(1) Control diet (fiber 14 g/day) (2) High cereal-fiber diet, HCF (fiber 43 g/day) (3) High protein diet, HP (28 E% protein, 14 g/day fiber) (4) Combined HCF and HP diet (23 E% protein, 26 g/day fiber)	↔ Dominant groups of gut bacteria ↔ Fecal acetate, propionate, butyrate, valerate Within groups: (3) ↑ Valerate	Within groups (2) ↑ Ins sensitivity (Euglycaemic hyperinsulinaemic clamps)

Significant differences (*p* ≤ 0.05) between intervention group(s) and the control group are shown with ↑ or ↓ while ↔ indicates no significant difference. When several the intervention groups are present, the results for each group are indicated with the number. Fasting values are shown, if not otherwise stated. The control group is referred to as (1). AT-IR: Adipose tissue insulin resistance, BMI: body mass index, F: Female, GLP-1: Glucagon-like peptide 1, Glu: Glucose, g: gram, HbA1c: Glycated hemoglobin A1c, HCF: High cereal-fiber diet, HP: High protein diet, HDL-C: HDL-Cholesterol, HOMA-IR: Homeostasis assessment model-insulin resistance, Ins: Insulin, IHTG: Intrahepatic triglycerides, IPE: Inulin-propionate ester, LDL-C: LDL-Cholesterol, M: Male, Matsuda ISI: Matsuda insulin sensitivity index, *n*: numbers, NEFA: Non-esterified fatty acids, TG: Triglycerides, TC: Total cholesterol.

**Table 4:** Fiber, gut microbiota, and metabolic regulation in people with metabolism-related disorders. Significant results are indicated by an up/down arrow.

Study	Subject Characteristics	Study Design	Intervention	Changes Related to Gut Microbiota	Changes Related to Metabolic Regulation
De Faria Ghetti et al., J Gastrointestin Liver Dis, 2019 [42]	*n* = 40, NASH, BMI 31, 50.6 y (Control), 48.3 year (DIET), M/F	3 months Parallel	(1) Control group (nutritional orientation) (2) The DIET group (fiber 30 g/day + nutritional orientation)	Within groups: (2) ↑ Density of total microorganisms (1) ↓ Bacteroidetes, Verrucomicrobiales	↓ Ins, HOMA-IR, ↓ TC Within groups: (2) ↓ Glu, HOMA-IR, TC, TG ↓ TC, LDL-C, TG
Velikonja et al., Ana in Microbiome, 2019 [43]	*n* = 43, MetS, BMI not reported, 50.9 year, M/F	4 weeks Parallel	(1) Control (Bread without b-glucan) (2) Bread with b-glucan (6 g/day)	Within groups: (2) ↓ Microbial diversity and richness, Higher basal abundance of *Bifidobacterium* spp and *Akkermansia municiphila* within the intervention group (2) ↑ Fecal propionate (1) ↓ Fecal acetate	Within group: (1) (2) ↔ Glu and Ins after OGTT (2) ↓ TC (1) (2) ↔ LDL-C, HDL-C, TG
Zhao et al., Science, 2018 [44]	*n* = 43, T2D, BMI not reported, 35–70 year, M/F	84 days Parallel	(1) Usual diet according to the Chinese Diabetes Society + acarbose (fiber 16.1 g/day) (2) Wholegrains, traditional Chinese medicinal foods and prebiotics + acarbose (fiber 37.1 g/day)	↑ SCFA-producing strains Within groups: (1) (2) ↓ Gene richness, tended to be higher in the (2) than in (1) this trend was associated with better clinical outcomes in group (2)	↓ HbA1c, Glu, GLP-1 AUC
Connolly et al., Front Microbiol, 2016 [45]	*n* = 30, mildly hypercholesterolemia or glucose-intolerant, BMI 26, 42 year, M/F	2 × 6 weeks crossover	(1) Non-whole-grain breakfast cereals 45g/day (fiber 3.0 g/day, no β-glucan) (2) Whole-grain oat granola 45 g/day (fiber 6.3 g/day and 2.9 g/day β-glucan)	↑ *Bifidobacterium* spp., *Lactobacillus* spp., total bacterial count ↔ Acetate, Propionate, Butyrate Within groups: (1) ↓ *Bifidobacterium* spp., total bacterial count (2) ↑ *Bifidobacterium* spp., *Lactobacillus* spp., total bacterial count	↔ Glu, Ins, HOMA-IR, QUICKI ↓ TC, LDL-C ↔ HDL-C. TG Within groups: (1) ↑ TC (2) ↓ TC
Pedersen et al., Br J Nutr, 2016 [46]	*n* = 29, T2D, BMI 30, 42–65 year, M	12 weeks Parallel	(1) Maltodextrin (5.5 g/day) (2) Galacto oligosaccharides (5.5 g/day)	↔ Bacterial abundance or diversity Within groups: (2) ↑ Diversity Shannon indices Correlations: ↓ *Veillonellaceae* and Glu response	↔ Glu, Ins, C-peptide (fasting or response IVGTT) ↔ TC, LDL-C

Significant differences (*p* ≤ 0.05) between the intervention group(s) and the control group are shown with ↑ or ↓ while ↔ indicates no significant difference. When several intervention groups are present, the results for each group are indicated with the number. Within-group changes are indicated with numbers. Fasting values are shown, if not otherwise stated. The control group is referred to as (1). F: Female, GLP-1: Glucagon-like peptide 1, Glu: Glucose, g: gram, HbA1c: Glycated hemoglobin A1c, HDL-C: HDL-Cholesterol, HOMA-IR: Homeostasis assessment model-insulin resistance, iAUC: Incremental Area Under the Curve, Ins: Insulin, IPE: Inulin-propionate ester, IVGTT: Intravenous glucose tolerance test, LDL-C: LDL-Cholesterol, M: Male, Matsuda ISI: Matsuda insulin sensitivity index, MetS: Metabolic syndrome, *n*: numbers, NASH: Non-alcoholic steatohepatitis, NEFA: Non-esterified fatty acids, OGTT: Oral glucose tolerance test, QUICKI: Quantitative insulin check, TG: Triglycerides, TC: Total cholesterol, T2D: Type 2 diabetes.
